# Transition from Seeds to Seedlings: Hormonal and Epigenetic Aspects

**DOI:** 10.3390/plants10091884

**Published:** 2021-09-11

**Authors:** Galina Smolikova, Ksenia Strygina, Ekaterina Krylova, Tatiana Leonova, Andrej Frolov, Elena Khlestkina, Sergei Medvedev

**Affiliations:** 1 Department of Plant Physiology and Biochemistry, St. Petersburg State University, 199034 St. Petersburg, Russia; s.medvedev@spbu.ru; 2 Postgenomic Studies Laboratory, Federal Research Center N.I. Vavilov All-Russian Institute of Plant Genetic Resources, 190121 St. Petersburg, Russia; k.strygina@vir.nw.ru (K.S.); e.krylova@vir.nw.ru (E.K.); khlest@bionet.nsc.ru (E.K.); 3 Department of Bioorganic Chemistry, Leibniz Institute of Plant Biochemistry, 06120 Halle (Saale), Germany; tanyaleonova2710@gmail.com (T.L.); Andrej.Frolov@ipb-halle.de (A.F.); 4 Department of Biochemistry, St. Petersburg State University, 199034 St. Petersburg, Russia

**Keywords:** desiccation tolerance, DNA methylation, epigenetics, germination, histone modification, hormonal regulation, miRNA, post-germination, seeds

## Abstract

Transition from seed to seedling is one of the critical developmental steps, dramatically affecting plant growth and viability. Before plants enter the vegetative phase of their ontogenesis, massive rearrangements of signaling pathways and switching of gene expression programs are required. This results in suppression of the genes controlling seed maturation and activation of those involved in regulation of vegetative growth. At the level of hormonal regulation, these events are controlled by the balance of abscisic acid and gibberellins, although ethylene, auxins, brassinosteroids, cytokinins, and jasmonates are also involved. The key players include the members of the LAFL network—the transcription factors LEAFY COTYLEDON1 and 2 (LEC 1 and 2), ABSCISIC ACID INSENSITIVE3 (ABI3), and FUSCA3 (FUS3), as well as DELAY OF GERMINATION1 (DOG1). They are the negative regulators of seed germination and need to be suppressed before seedling development can be initiated. This repressive signal is mediated by chromatin remodeling complexes—POLYCOMB REPRESSIVE COMPLEX 1 and 2 (PRC1 and PRC2), as well as PICKLE (PKL) and PICKLE-RELATED2 (PKR2) proteins. Finally, epigenetic methylation of cytosine residues in DNA, histone post-translational modifications, and post-transcriptional downregulation of seed maturation genes with miRNA are discussed. Here, we summarize recent updates in the study of hormonal and epigenetic switches involved in regulation of the transition from seed germination to the post-germination stage.

## 1. Introduction

Seed development is a critical step in the ontogenesis of higher plants. Obviously, it is crucially important in terms of plant survival and successful reproduction. Thereby, mature seeds are typically highly dehydrated and can be considered as units of dispersal and survival during the periods of unfavorable environmental conditions [1,2,3]. On the other hand, for successful propagation, germination of seeds needs to be associated with the periods of optimal water and temperature regime. To adjust growth of seedlings to environmental conditions, spermatophyte plants evolved an ability to control the time of germination [4]. This ability relies on the phenomenon of dormancy, i.e., a period or temporal inhibition of plant growth, which impacts on the prevention of germination under unfavorable conditions [5,6]. Thus, release from seed dormancy is controlled by such environmental factors as light, temperature, and duration of dry storage, whereas hormonal regulation, genetic, and epigenetic factors impact essentially on this phenomenon [7,8,9,10,11,12].

The network of four master regulators, which is usually referred to as LAFL, i.e., LEAFY COTYLEDON1 (LEC1), ABSCISIC INHIBITOR3 (ABI3), FUSCA3 (FUS3), and LEC2, is directly involved in coordination of seed maturation and inhibition of seed germination [10,13]. Thus, LAFL acts as a positive regulator of seed maturation genes, whereas the players involved in inhibition of germination are only partly addressed so far. The switch of the developmental program from maturation to germination is accompanied with suppression of LAFL genes and activation of those involved in vegetative growth [14,15]. It was shown that expression of LAFL genes is negatively regulated by two transcriptional repressors, namely HIGH-LEVEL EXPRESSION OF SUGAR INDUCIBLE GENE2 (HSI2) and HSI2-LIKE1 (HSL1), also often referred to as VP1/ABI3-LIKE1 (VAL1) and VAL2, respectively [16]. The mechanisms behind the germination-related repression of the LAFL transcriptional network by HSI2 and HSL1 rely on modification of chromatin in particular, the chromatin remodeling complexes POLYCOMB REPRESSIVE COMPLEX1 and 2 (PRC1 and PRC2) [17,18,19,20], as well as PICKLE (PKL) and PICKLE-RELATED2 (PKR2) proteins [21,22,23]. Alterations in chromatin structure underlying changes in gene activity can be related to post-translational modifications of histones—N-terminal acetylation, side-chain methylation, phosphorylation, ubiquitination, and SUMOylation. Thereby, acetylation and methylation of histone H3 at its lysine residues are the most critical contributors in epigenetic regulation of gene expression.

Importantly, the patterns of histone modifications, also referred to as histone code, serve as specific markers for recruitment of further players of the chromatin remodeling machinery [24,25,26]. Specifically, it was shown that the chromatin remodeling factor PKL directly blocks the *DELAY OF GERMINATION1* (*DOG1*) gene—the key player in induction and maintenance of seed dormancy [27,28,29]. The authors found that PKL physically interacted with LUX ARRHYTHMO (LUX), a player of the evening complex involved in the circadian clock. LUX was shown to bind directly to a specific coding sequence of DOG1. The loss of function of either PKL or LUX resulted in decreased levels of trimethylation at K27 in histone H3 in the *DOG*1 locus. Zha et al. suggested that LUX binds directly to a specific DNA sequence of *DOG*1 and recruits PKL to the *DOG*1 locus through their physical interaction [28]. This interaction increases the levels of the trimethyllysine at K27 in histone H3 (H3K27me3), representing the part of chromatin bound to DOG1, thereby repressing transcription of this gene and leading to reduced levels of seed dormancy [28].

Thus, before plants enter the vegetative phase upon germination, the rearrangements of signaling pathways and switching of gene expression programs occur. However, despite their importance for understanding the following plant development, the molecular mechanisms behind these switches remain mostly unknown. Therefore, here, we highlight the recent advances and provide a comprehensive overview of the recently reported experimental data, providing access to understanding of epigenetic and hormonal regulation of the seed-to-seedling transition.

## 2. Stages of Seed Germination 

**Figure 1:**
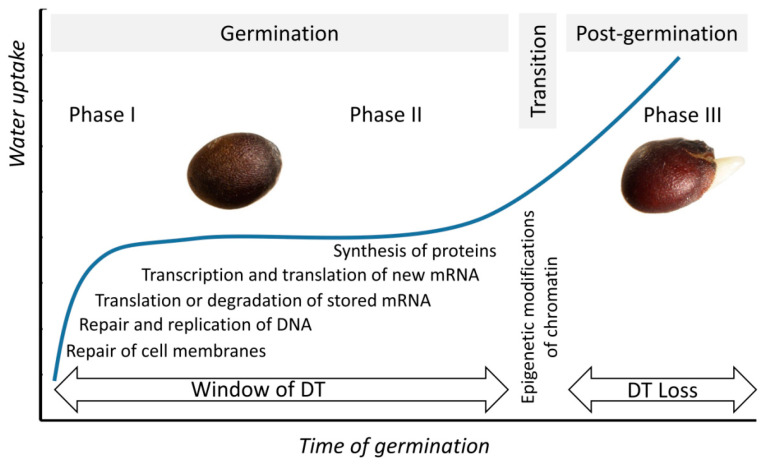
Time course of seed germination. The overall germination times vary from hours to weeks, depending on plant species and environmental conditions. Phase I is characterized by rapid water uptake, accompanied with enhanced hydration of macromolecules, activation of respiration, repair of membranes, mitochondria, and DNA. Phase II is characterized by mobilization of reserves, translation of stored mRNAs, transcription and translation of newly synthesized mRNAs, and activation of protein biosynthesis. The radicle protrusion is considered as the beginning of Phase III. Epigenetic changes (methylation of DNA, as well as trimethylation, ubiquitination, and acetylation of histones), occurring in this phase, result in silencing of the genes related to seed maturation and triggering expression of the genes responsible for vegetative growth of seedlings. “Window of DT (desiccation tolerance)” can be defined as the part of the overall germination period, when seeds can be dried back to their original water contents without a decrease of their viability. Transition from germination to the post-germination stage corresponds to loss of DT.

In general, the overall process of seed germination can be divided into two principal periods, which are often referred to as phases I and II [2,30,31,32] (Figure 1). Imbibition represents the first phase, which is characterized with fast water uptake, hydration, and softening of the seed coat due to degradation of seed coat polymers. Thereby, seed water content rapidly increases due to enhanced hydration of macromolecules. Recently, Dorone et al. identified the prion-like protein FLOE1, which forms condensates during imbibition and attenuates germination under water-limiting conditions in a dose-dependent manner [33]. Imbibition is accompanied with activation of respiration and, therefore, ROS production. On the other hand, this phase features repair of membranes, mitochondria, and DNA. In turn, phase II is characterized by activation of the principal metabolic processes associated with seed germination—mobilization of reserve substances, translation of stored mRNAs, transcription and translation of newly synthesized mRNAs, processing of proteins, and their co- and post-translational modification. It is important to note that both phases I and II are critical for maintaining seed viability. Thus, enhanced respiration and water uptake result in dramatic upregulation of ROS production. To avoid accumulation of molecular damage, repair of DNA and proteins is enhanced during phases I and II [31]. 

With respect to desiccation tolerance (DT), the seeds of vascular plants can be classified into orthodox and recalcitrant types [34,35,36,37]. During the last stages of maturation, the seeds of orthodox type acquire DT, which allows them to sustain unfavorable germination conditions in the metabolically inactive dry state [2,11,38,39]. The inhibited metabolic processes are resumed during germination afterwards. This ultimately results in the loss of desiccation tolerance at the stage of radicle protrusion [40,41] (Figure 1), which can be considered as the beginning of phase III—post-germination. Importantly, the part of the overall germination process before this time point is usually referred to as the “window of DT”, i.e., the period when germinating seeds can be dried to their original water contents without loss of seed viability and any deterioration of their quality [42,43]. Already in the last decade, Buitink et al. showed that *Medicago truncatula* L. seeds with a radicle length of up to 1 mm can sustain a short-term dehydration, whereas the DT of the seeds was completely dependent on the degree of applied osmotic stress when the radicle length achieved 2.7 mm [40]. This fact illustrates the effect of osmopriming, which can extend a species-specific DT window [40,44]. It is known that DT of germinated *Arabidopsis thaliana* (L.) Heynh. seeds was, to a large extent, dependent on the presence of ABA in the germination medium [43,45,46]. Similar to PEG, ABA can extend the period of DT. The corresponding time window, when seeds are responsive to the introduction of ABA in medium, was described by Lopez-Molina et al. [47]. 

Phase III is characterized by progressing division and elongation of radicle cells, degradation of endosperm, and radicle protrusion [2,30]. Thereby, loosening of the cell wall in the hypocotyl region (which underlies elongation of radicle cells) is mediated by ROS. The main source of the oxidative burst accompanying radicle elongation is the superoxide anon radical generated by NADPH oxidases of the plasma membrane [48,49]. The transition from phase II to phase III can be treated as the transition from seed to seedling.

## 3. Hormonal Regulation of Seed Germination

### 3.1. ABA and GAs Signaling

The release from dormancy and acquiring the germination ability are defined by the balance of phytohormones, with a strong contribution of environmental factors such as temperature, water availability, and light [50,51,52,53]. Thereby, ABA and GAs act as the main endogenous regulators, which control seed dormancy and germination in an antagonistic manner [6,8,50,54,55,56,57,58,59]. Specifically, ABA promotes seed dormancy and inhibits germination, whereas GAs, in contrast, disrupt seed dormancy and trigger germination. At the early stages of embryogenesis, low ABA contents, required for embryo development, are established by the maternal ovarian tissues, whereas the further developmental stages (seed maturation and dormancy) are controlled by ABA synthesized in the seed itself [54,57,60]. Finally, after the start of imbibition, the embryo switches to the synthesis of GAs. These hormones are transported to the aleurone layer, where they trigger expression of the genes encoding α-amylases and proteases [61]. 

Recently, a unique DOG1-dependent ABA signaling pathway was characterized in Arabidopsis seeds [27,62,63,64]. DOG1 is a master regulator of primary dormancy, onset of which follows seed maturation [29]. It was found that DOG1 interacts with negative regulators of ABA signaling and seed dormancy—phosphatases ABA-HYPERSENSITIVE GERMINATION 1 and 3 (AHG1 and AHG3)—preventing their involvement in the release of seed dormancy [65,66].

AHG1 and AHG3 belong to the group A type 2C protein phosphatases (PP2Cs) suppressing sucrose-non-fermenting-related kinases (SnRK2), which positively regulate the activity of the transcription factors ABI3 and ABI5 [65,66]. Binding of DOG1 to AHG1 and/or to AHG3 triggers the release of SnRK2, which phosphorylates ABI5 and ABI3 [11]. Thus, the DOG1-dependent signaling pathway results in the inhibition of PP2C family members, which, in turn, suppress the expression of ABA-responsive genes [67].

Developing seeds of some higher plants contain photochemically active chloroplasts and chlorophylls, which are typically destroyed at the late maturation stage [68,69,70,71]. Shanmugabalaji et al. showed that GAs control the biogenesis of chloroplasts in developing seedlings [72]. When the GA contents in germinating seeds are low, their negative regulator DELLA (RGL2) can accumulate in tissues [73]. DELLA blocks the conversion from pro-plastids to chloroplasts by promoting the degradation of TRANSLOCASE OF CHLOROPLAST159 (TOC159) via the ubiquitin/proteasome system [72]. TOC159 is known to mediate recognition of pre-protein and regulates its transport into plastids [74]. The increase of GA contents results in degradation of DELLA. This makes TOC159 available for assembly into the TRANSLOCON ON THE OUTER CHLOROPLAST MEMBRANE (TOC) complex, which accomplishes the import of photosynthesis-associated proteins into the chloroplast [72].

According to the current state of the knowledge, hydrogen peroxide (H_2_O_2_) acts as the master ROS-related secondary messenger involved in regulation of seed germination [75,76,77,78]. Specifically, it alters the balance between ABA and GAs by promoting the expression of *CYP707A2* genes involved in ABA degradation, and increasing the expression of *GA3ox1* genes involved in GA biosynthesis [79,80]. Not less importantly, enhancement of ROS generation triggers activation of GA signaling and promotes inactivation of ABA signaling [81]. During the recent decade, special attention was paid to the cross-talk between ROS- and phytohormone-mediated signaling pathways during seed germination [77]. Thus, Bailly et al. proposed that increased tissue ROS levels might shift the ABA/GA ratio in favor of GAs, i.e., the phytohormones triggering germination [81]. 

### 3.2. Role of Ethylene, Cytokinins, Brassinosteroids, IAA, and Jasmonates 

Besides ABA and GA, other hormones, such as ethylene [82,83,84,85], cytokinins [8,86], brassinosteroids [8,87], IAA [8,55,88,89], and jasmonates [84,90], are also involved in the control of seed dormancy and germination. 

Ethylene interferes with ABA- and GA-related signaling pathways, promoting seed germination in numerous species [8,83,91]. On the one hand, ethylene acts as an ABA antagonist by suppressing the regulation of ABA metabolism and signaling [6,8,92]. In some species (e.g., Brassicaceae), ethylene prevents the inhibitory effects of ABA by facilitating endosperm rupture of germinating seeds [6,60,92,93]. On the other hand, ethylene impacts the GA biosynthesis via modulation of *GA3ox* and *GA20ox* gene expression and GA signaling via DELLA proteins [94].

Cytokinins can also promote germination at the signaling level, acting as antagonists of ABA [86,95,96]. Specifically, ABA triggers downregulation of *Arabidopsis Response Regulator*s (*ARR*s), a family of genes induced by cytokinins during seed germination and cotyledon greening [86]. Among the type-A *ARR* family members, expression of *ARR*6, *ARR*7, and *ARR*15 was reported to be upregulated in ABA-deficient mutants. In turn, *ARR*6, *ARR*7, and *ARR*15 attenuated the ABA-mediated inhibition of germination. Application of exogenous ABA suppressed the type-A ARRs in Arabidopsis seeds and seedlings. Among the type-A *ARR* family members, expression of *ARR*6, *ARR*7, and *ARR*15 was upregulated in ABA-deficient mutants. In turn, *ARR*6, *ARR*7, and *ARR*15 proved to be negative regulators of ABA-mediated inhibition of germination. ABSCISIC ACID-INSENSITIVE4 (ABI4) plays the key role in ABA and cytokinin signaling by inhibiting transcription of type-A *ARR*s [86]. The ABI4 is a crucial regulator of the ABA signaling pathway during seed development, providing functional interactions between ABA and other hormones [86,97,98,99,100]. ABI4 modulates ABA and GA metabolism by targeting *CYP707A1, CYP707A2,* and *GA2ox7*. It is involved in the suppression of ethylene biosynthesis by targeting *ACS4* and *ACS8* [98,100]. A high level of ABA in dormant Arabidopsis seeds enhances the transcriptional activity of *ABI4*. In the presence of high ABA content, this factor blocks induction of ARR6/7/15, resulting in the suppression of cytokinin responses. After completion of germination, cytokinins stimulate accumulation of ARR4/5/6 [86].

Brassinosteroids (BRs) are ABA antagonists and, like GAs, can promote seed germination by enhancing the growth potential of the embryo [7,58,101,102,103]. In Arabidopsis, the BRs biosynthetic mutant *det2-1* and the BRs responsive mutant *bri1-1* were shown to be more sensitive to inhibition of ABA than the wildtype [101]. This observation indicates that the pathways of ABA and BR signaling might work as antagonistic regulators of seed germination. Recently, Sun et al. revealed that BRs signaling represses the accumulation of PIN-LIKES (PILS) proteins at the endoplasmic reticulum, thereby increasing nuclear abundance and signaling of auxin [104].

Auxin maintains a high level from fertilization to seed maturation by PIN carriers [105]. Auxin transport from endosperm is regulated by AGAMOUS-LIKE62 (AGL62), which is specifically expressed in the endosperm [106]. Auxins have recently emerged as essential players which modulate (in concert with ABA) different cellular processes involved in seed development, dormancy, and longevity [107,108,109]. Thereby, ABI3 appeared to be critical for cross-talk between auxin and ABA signaling [107,109]. In developing Arabidopsis embryos, the longevity-associated genes with promoters enriched in IAA response elements and ABI3 were induced by auxin [109], but the effect of exogenous auxin treatment was abolished in *abi3-1* mutants. 

Recently, Hussain et al. showed that the auxin signaling repressor Aux/IAA8 accumulates and promotes seed germination. The IAA8 loss-of-function mutant iaa8-1 exhibited delayed seed germination. IAA8 was shown to suppress transcription of ABI3, a negative regulator of seed germination. Accumulation of IAA8 promotes seed germination by inhibiting AUXIN RESPONSE FACTOR (ARF) activity, which is accompanied by downregulating *ABI3* gene expression [89].

Treatment of wheat (*Triticum aestivum* L.) with *methyl jasmonate* inhibited expression of the ABA biosynthesis-related gene, *Ta9-cis-EPOXYCAROTENOID DIOXYGEN-ASE*1 (*TaNCED*1), which resulted in a decrease of seed ABA contents [110]. However, in Arabidopsis, jasmonate precursor (12-oxo-phytodienoic acid) inhibited seed germination, indicating that the role of jasmonates in dormancy varies between the species [111]. Xu et al. found that cold-induced germination of dormant embryos correlated with a drop of ABA contents and an increase of jasmonic acid (JA) levels, along with expressional enhancement of JA biosynthesis [90]. It was shown that the cold-induced increase in JA contents was required for the release of seed dormancy [90]. The increase of JA levels was, at least partly, mediated by the repression of two key ABA biosynthesis genes—*9-cis-EPOXYCAROTENOID DIOXYGENASE 1* and *2* in bread wheat *Triticum aestivum* L. (*TaNCED1* and *TaNCED2*). These genes encoded for 9-*cis*-epoxycarotenoid dioxygenase, catalyzing oxidative cleavage of *cis*-epoxycarotenoids—a critical step in ABA biosynthesis in higher plants [112].

### 3.3. The Effects of Light and Temperature

Light is a critical regulator of seed germination, especially for light-loving species with small seeds [113,114]. For most of the higher plants, seed germination is triggered by red and repressed by far red parts of the spectrum [54,115]. While far red light increases the tissue levels of ABA and suppresses GA biosynthesis, red light has an opposite effect [116,117]. Light is the key environmental signal, and phytochromes redundantly affect seed germination, with phytochrome B (PhyB) playing the major role in this process [114,116]. During the early stages of seed imbibition, Phy B mediates the R/FR photo reversible response to trigger germination. Phytochrome A (PhyA) is directly involved in irreversible photoinduction of seed germination via irradiation with low-fluence light in a broad spectral band from ultraviolet-A to the far red region of the spectrum [118,119].

The key element of the seed light-dependent signal transduction pathways is phytochrome-interacting factor 1 (PIF1), also known as PIF3-LIKE 5 (PIL5), which is known to strongly suppress seed germination in the dark via modulating the expression of GA- and ABA-related genes [114,120]. Indeed, PIF1 inhibits germination by suppressing GA biosynthesis and GA-related signaling, with a simultaneous activation of the ABA biosynthesis and signaling [116]. This inhibition is controlled by PhyB. Activation of this protein by red light leads to the degradation of PIF1. On the other hand, inactivation of PhyB by far red light results in stabilization of PIF1. Thus, light acts as a switch, affecting the balance between ABA and GA metabolism via a phytochrome-mediated mechanism, based on the PIF1 degradation and stabilization. 

The temperature is another critical environmental cue affecting seed dormancy and germination timing [9,121]. Thus, application of low temperatures during seed imbibition typically stimulates seed germination (so-called stratification), whereas high temperatures inhibit it [1,117]. Cold stratification was shown to interrupt seed dormancy and to enhance germination by modulation of the balance between ABA and GAs. Recently, Yamauchi et al. found that a subset of GA biosynthesis genes was upregulated in response to low-temperature treatment [122]. This resulted in higher transcript abundances of GA-inducible genes in imbibed Arabidopsis seeds and increased tissue levels of bioactive GAs. On the other hand, ABA metabolism and signaling also underlie the release of seed dormancy after cold stratification. During cold imbibition, ABA seed contents decrease and the expression of ABA-responsive genes changes [32]. 

## 4. Epigenetic Mechanisms of the Seed-to-Seedlings Transition

All the major epigenetic mechanisms, which are generally known in eukaryotes to date, were successfully confirmed in plants [26,123,124]. Thus, DNA methylation, post-translational modification of histones, and interaction with non-coding RNAs provide a multifactorial and robust basis for epigenetic regulation of plant development and adaptation [125,126,127,128]. Thereby, stable allelic epigenetic inheritance efficiently complements the hereditary role of DNA, representing an additional molecular mechanism underlying practically unlimited diversity [129].

### 4.1. DNA (de)Methylation

Generally, DNA methylation represents a covalent modification of the cytosine base, which is typically associated with the dinucleotide consensus CG. However, in plants, in contrast to other organisms, DNA methylation can also occur at cytosines localized to CHG and CHH consensus sequences, where H is A, C, or T (Bird, 1986; Finnegan et al., 1998). In coding sequences, methylation most commonly occurs at CG sites, while non-CG methylation (CHG and CHH) is much less common [130]. In angiosperms, CG methylation accounts for more than 50% of the total cytosine methylation [131]. However, independently from the specific consensus sequence, the overall methylation patterns of genomic DNA vary essentially among plant species. Thereby, the heterogeneity of CHG and CHH methylation is higher in comparison to the modification patterns, characteristic for the CG sites [131,132,133].

In plants, the occupation of potential methylation sites decreases upstream of the transcription start site (TSS) and around the transcription termination site (TTS), while the degree of methylation inversely correlates with gene expression levels in promoter regions [130,134]. On the other hand, moderately expressed genes are methylated in gene bodies [134,135]. Thus, in the Arabidopsis genome, 73% of DNA methylation sites are located in exons, whereas only 8% of them can be found in putative promoter regions, 3% in introns, and 16% in extragenic regions [51].

It is generally agreed that DNA methylation is an epigenetic modification underlying the silencing of transposable elements (TE) and directly involved in gene expression regulation. It plays a critical role in plant growth and development [130]. Accordingly, both seed development and germination are accompanied by dynamic reconfiguration of DNA methylation [136,137]. DNA methylation is represented by two forms—maintenance methylation and de novo methylation [138]. Maintenance methylation assumes recognition of the methylation marks on the DNA parental strand and transfers new methylation to the daughter strands after DNA replication. During de novo methylation, transfer of methyl groups to cytosines of DNA occurs independently from their previous methylation by DRM2, with the participation of the RNA-directed DNA methylation (RdDM) pathway [139,140,141]. It is de novo methylation that is involved in the rearrangement of methylation patterns during differentiation processes. Several distinct DNA methyltransferases are involved in generation (de novo) and subsequent maintenance of DNA methylation at three sequence contexts.

In Arabidopsis, DNA METHYLTRANSFERASE 1 (MET1) is the major enzyme involved in maintaining CG methylation [142,143,144]. In contrast, methylation at CHH and CHG sites typically relies on activities of two enzymatic systems—DNA CHROMOMETHYLASE 2 and 3 (CMT2 and CMT3) and DOMAINS REARRANGED METHYLTRANSFERASES 1 and 2 (DRM1 and DRM2) [145,146,147,148,149,150].

Recently, it was shown that precocious germination of *Solanum lycopersicum* L. seeds could be promoted by silencing of MET1 [151]. This was associated with a decrease in the contents of mRNAs encoding 9-*cis*-epoxycarotenoid-dioxygenase—a key enzyme of ABA biosynthesis.

As repression of TEs is required for stability of the plant genome, they are typically located in transcriptionally inactive regions [152]. Thus, potential methylation sites in long and gene-distal TEs are the typical targets for both CMT2 and CMT3 [153]. Maintenance of CHH methylation at short, gene-proximal TEs as well as at the edges of long TEs requires the mechanism of RNA-dependent DNA methylation (RdDM), which involves DNA-dependent RNA polymerases IV and V (Pol IV and Pol V) [152,154]. This pathway involves two main steps: an upstream small interference RNA (siRNA) biogenesis phase and a downstream methylation targeting phase. Pol IV produces short precursor RNAs that are processed into 24 nt small interfering RNAs (siRNAs) by a Dicer-like endonuclease 3 (DCL3) and further loaded into ARGONAUTE 4 (AGO4), forming AGO4-siRNA complexes [155,156]. Pol V produces non-coding RNA transcripts that are proposed to act as a scaffold at sites of DNA methylation [157]. These scaffold transcripts are bound by Pol IV-dependent 24 nt siRNAs that recruit DRM1 and DRM2 to maintain DNA methylation [154,158].

**Figure 2:**
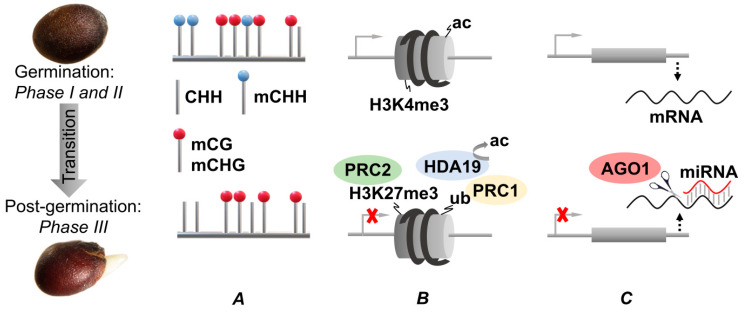
Epigenetic modifications accompanying the transition from germination to the post-germination stage of plant development. (**A**) Methylation of cytosines in DNA. CHH methylation (mCHH) is gradually lost during germination. In contrast, CG and CHG methylation (mCG and mCHG) mostly remain stable. (**B**) Histone post-translational modifications. The genes involved in seed maturation and dormancy are repressed by trimethylation of K27 in histone H3 by PRC2 and ubiquitination at K119 of histone H2A by monoubiquitin ligase PRC1. The repression of the target gene is initiated by recruitment of histone deacetylase 19 (HDA19) by VAL proteins to remove histone acetylation marks and PRC1 to incorporate the H2A ubiquitine marks. Upon its onset, stable repression of the target genes can be constantly maintained by PRC2-mediated trimethylation of K27 in histone H3. (**C**) Post-transcriptional downregulation of target genes. miRNAs (red wavy line) block the expression of the genes involved in development and dormancy genes by cleavage of their mRNAs with Argonaute1 (AGO1) proteins.

DNA methylation patterns have a clearly dynamic character and are continuously changing during plant seed development [159,160,161,162]. Thus, the occupancy of the CHH methylation sites remarkably increases from the early to the late stages of seed development and gradually decreases later on during germination. Thereby, both RdDM and CMT2 are responsible for CHH methylation in developing seeds, although both these enzymes lose their activity upon germination [136,163]. In soybean (*Glycine max* (L.) Merr.), DNA methylation in the CHH context increased from 6% at the early stage of seed development to 11% at the late stage [161]. Thus, the dynamics of soybean and Arabidopsis seed methylomes were clearly similar, i.e., the levels of CHH methylation gradually increased during seed development from fertilization to onset of dormancy in all parts of the seeds [164]. In contrast to the CHH sites, the patterns of CG and CHG methylation remain, to a large extent, unchanged over the whole period of seed development (Figure 2A) [136,161,162,163,164,165]. 

Later, during the seed maturation step, the levels of CHH methylation in TEs decrease drastically [163]. For example, it was shown for the Arabidopsis quadruple mutant *ddcc* (*drm1 drm2 cmt2 cmt3*), which was deficient in all methyltransferases required for all types of non-CG methylation [164]. The authors found that more than 100 TEs were transcriptionally derepressed in *ddcc* seeds. This might indicate reinforcement of TE silencing in developing seeds upon the upregulation of cytosine methylation in the CHH consensus sequence [164]. Thus, the proposed mechanism might underlie the constantly silent state of TEs, which, therefore, do not inactivate genes essential for seed development.

Multiple genes involved in seed development and germination are located in hypomethylated regions of the genome, known as DNA methylation valleys (DMVs, [166]). The DNA methylation status of these regions remains unchanged during the whole period of seed development, from fertilization to germination. Indeed, several genes encoding the enzymes of hormone biosynthesis (e.g., gibberellic acid oxidase *GmGA20Ox2*, *GmGA3Ox1, AtGm20Ox2,* and *AtGA3Ox1*), storage proteins (e.g., *GmGlycinin1, AtCruciferin1*), and some transcriptional regulators, are located within hypomethylated regions of the soybean and Arabidopsis genomes [166]. DMVs constitute an important part of the soybean seed genome, which does not vary significantly in the context of methylation status during seed development and early germination [166]. Moreover, genome regions hypomethylated during the plant lifecycle are enriched in genes encoding TFs, as well as in the genes critically impacting on seed formation (*LEC1*, *ABI3*, and *FUS3*) [10,166].

Seed germination is accompanied by silencing of the genes involved in seed development and activation of those controlling vegetative growth, mostly associated with cell division and cell wall organization. These genes are typically methylated throughout seed development and are later demethylated during germination [136,163]. Hypermethylation of genes in germinating seeds is reprogrammed mainly by passive CHH demethylation. 

Demethylation of plant DNA can be either passive or active. Passive demethylation occurs when the new chain in the replicated DNA molecule is not involved in maintaining methylation. In this case, only the old (maternal) chain appears to be methylated [26]. In contrast, active demethylation relies on the activity of demethylases represented in Arabidopsis by four enzymes: DEMETER (DME), REPRESSOR OR SILENCING 1 (ROS1), DEMETER-LIKE 2 (DML2), and DEMETER-LIKE 3 (DML3) [167,168].

### 4.2. Modification of Histones

Different types of histone post-translational modifications have been described to date in the context of epigenetic regulation of seed development and germination. Thus, alterations in chromatin structure leading to gene expression changes can be underlined, at least by acetylation, methylation, phosphorylation, ubiquitination, and SUMOylation of histones [24,25,26]. These modifications play an important role in control of seed maturation, dormancy, and germination [10,62]. Thereby, the patterns of histone modifications (so-called histone code) serve as the marks for attachment of other proteins, involved in remodeling of chromatin. Methylation and acetylation of lysine residues in histone H3 directly affect the expression of associated genes. 

Thus, the cycles of histone acetylation and deacetylation are important elements in the regulation of genome activity [169]. Acetylation of lysine side chains affects the overall positive charge of histones and charge distribution on their surface [170,171]. It ultimately affects the interaction of histones with negatively charged phosphate groups of DNA and results in de-condensation of chromatin. The resulted relaxed structure is associated with its higher transcriptional activity. This relaxation can be reversed by the activity of deacetylases [172] (Figure 2B).

Trimethylation at K4 and K27 of histone H3 (H3K4me3 and H3K27me3), leading to activation or suppression of gene expression respectively, represent the most well-characterized examples of site-specific post-translational modifications of histones (Figure 2B). H3K27me3 plays a critical role in the regulation of genes involved in plant developmental control [10,26].

In plants, H3K27me3 is found in transcriptionally inactive regions of promoters and in transcribed regions of individual genes, whereas H3K4me3 is an antagonistic modification of histones in transcriptionally active regions [173]. Multiple DNA methylation valley (DMV) genes contain H3K4me3, H3K27me3, or bivalent marks that fluctuate during development [25,174]. The Arabidopsis H3K4me3 demethylases are also known as Arabidopsis trithorax (ATX) and Arabidopsis trithorax-related (ATXR) [175] (Table 1).

In mature embryos, *ABI*3 and *LEC*2 are associated with H3K4me3, which marks these genes as transcriptionally active. However, upon germination, these modifications are replaced by antagonistic H3K27me3, which results in transcriptional deactivation of these genes [10]. Recently, Chen et al. revealed that H3K27me3 demethylase RELATIVE OF EARLY FLOWERING6 (REF6) directly upregulates the expression of abscisic acid 8′-hydroxylase 1 and 3 (*CYP707A1* and *CYP707A3*) involved in ABA catabolism in seeds [176] (Table 1). 

Polycomb proteins form chromatin-modifying complexes that implement transcriptional silencing in higher eukaryotes. Thus, hundreds of genes can be silenced by Polycomb proteins, including dozens of those encoding crucial developmental regulators in organisms from plants to humans [177,178]. Gene suppression typically relies on the PRC1 and PRC2. Both PRC1 and PRC2 are represented by several families of related complexes, which target specific repressed regions [10]. Thus, PRC2 is responsible for the trimethylation at K27 of histone H3 [177,179], whereas PRC1 catalyzes mono-ubiquitination of K119 in histone H2A, yielding a transcriptionally inactive chromatin conformation. It is generally agreed that PRC2 is required for initial targeting of the genomic region to be silenced, whereas PRC1 impacts on stabilizing this silencing and underlies the cellular memory of the silenced region after cell differentiation. The activity of PRC2 in plants can be inhibited by treatment with 1,5-bis-(3-bromo-4-methoxyphenyl)-penta-1,4-dien-3-one, which affects both seed germination and radicle growth [179].

Although the PRC1 complexes differ significantly between animals and plants, some of their components, such as ring-finger proteins RING1 and BMI1, are rather conserved [180]. In Arabidopsis, the PRC1 core components, AtRING1 and AtBMI1, were shown to physically interact with the PHD domain H3K4me3-binding ALFIN1-like (AL) proteins. The loss of AL6 and AL7 by T-DNA insertion mutant analysis, as well as the loss of AtBMI1a and AtBMI1b, retards seed germination and causes transcriptional de-repression, accompanied by a switch of histone modification state from H3K4me3 to H3K27me3 [17]. Therefore, AL PHD-PRC1 complexes associated with histone H3 act as switchers from the H3K4me3-associated active to the H3K27me3-associated repressive transcription state of the genes involved in seed development.

In the regulatory pathways involved in control of seed development, maturation, and germination, transcription factors containing the B3 DNA-binding domain (DBD) play the key role [181]. The DBD is a highly conserved domain consisting of 100–120 amino acid residues, designated as B3, that was originally identified as the third basic region in the ABI3 and VP1 proteins [182]. Among them, the LAFL network of transcription factors is directly involved in the activation of seed maturation, whereas VAL (VP1/ABI3-LIKE) proteins suppress LAFL-related effects, i.e., initiation of germination and vegetative development [16,183]. As was already mentioned above, chromatin remodeling complexes PRC1 and PRC2 [17,18], as well as the PKL and PKR2 proteins [21,22], are involved in repression of the LAFL network of transcription factors during seed germination [160]. For example, during seed germination, LAFL genes are repressed by the Polycomb machinery via its histone-modifying activities: the histone H3 K27 trimethyltransferase activity of the PRC2 and the histone H2A E3 monoubiquitin ligase activity of the PRC1 [184,185,186,187] (Figure 2B). Specifically, VAL proteins recruit histone deacetylase 19 (HDA19) and PRC1 to the chromatin regions, which contain genes involved in regulation of development and dormancy (Table 1). Thereby, HDA19 removes histone acetylation marks, whereas PRC1 incorporates monoubiquitinated histone H2A (H2Aub) marks to initiate initial repression of the target gene [188]. Thus, the VAL proteins (which are required for the introduction of H2Aub gene marks in histone H3 molecules) appear to cause the initial repression of the seed development- and germination-related genes. Later on, this repression is maintained by PRC2-mediated trimethylation at H3K27 [188]. It is important to note that VAL1 was shown to interact with HDA19 and to repress LAFL gene expression during germination [189].

**Table 1:** A selected list of epigenetic modifications of histones involved in seed development and germination.

Enzymes	Function	Target Gene(s)	Trait Affected	Species	References
PRC2	Trimethylation of K27 on histone H3 (H3K27me3)	*LEC1, ABI3, FUS3, LEC2, DOG1*, repression	Germination	*A. thaliana*	[18,184,186,187]
HDA19	Removal of histone acetylation mark (H3K9ac)	*LEC1, ABI3, FUS3, LEC2, DOG1*, repression	Germination	*A. thaliana*	[10,160,189]
PRC1	Ubiquitination at K119 of histone H2A (H2Aub)	*LEC1, ABI3, FUS3, LEC2, DOG1*, repression	Germination	*A. thaliana*	[17,185,188]
ATXR	Trimethylation of K4 on histone H3 (H3K4me3)	*LEC1, ABI3, FUS3, LEC2, DOG*, etc., transcriptional activation	Seed development	*A. thaliana*	[17,175]
REF6	H3K27me3 demethylase	CYP707A1, CYP707A3, transcriptional regulation	ABA catabolism in seeds	*A. thaliana*	[176]

Two other factors, playing an important role in repression of the embryonic state, were identified in Arabidopsis: PICKLE (PKL), encoding for the putative chromatin-remodeling factor CHD3, and gibberellins. It was found that PKL acts throughout the seedling, repressing the expression of embryonic traits, and is required for GA-dependent responses in shoots [190].

### 4.3. miRNA-Target Modules

It is well-known that the plant genome contains both protein-coding and non-coding sequences [191,192]. Non-coding sequences are represented by regulatory non-coding RNAs—microRNAs (miRNAs), long non-coding RNAs (lncRNAs), short interfering RNAs (siRNAs), and circular RNA (circRNA) [193,194]. Small non-coding RNAs (sRNAs) are known as important regulators of gene expression, affecting almost all stages of the plant lifecycle [195,196]. The regulatory RNAs of this type act at the transcriptional and post-transcriptional levels and essentially impact on seed development and germination [160,197,198]. 

The major class of plant sRNAs is represented by miRNAs, which are involved in regulation of plant development at the post-transcriptional level (Figure 2C). The biogenesis of miRNAs is a multistep process, including transcription of miRNA genes, processing of primary miRNAs, and loading of mature miRNAs into ARGONAUTE (AGO) proteins to form the miRNA-induced silencing complex (miRISC). Plant miRNAs are involved in multiple regulatory mechanisms, including mRNA cleavage, repression of translation, and DNA methylation [193,196,199,200].

The tissue contents of individual miRNAs change dynamically throughout the whole stages of seed development and germination. Thereby, their abundance correlates well to the phases of seed development, maturation, and germination [165,197]. Thus, miRNAs block the expression of the genes involved in control of development and dormancy via cleavage of mRNA by AGO1 proteins [193] (Figure 2C). Comprehensive analysis of miRNAs in canola seeds showed that miR156 is involved in regulation of the transition to germination [201] (Table 2). It was also shown that DOG1 affected the levels of miR156 and miR172 and could therefore regulate seed dormancy in lettuce [202]. Thereby, suppression of the DOG1 expression enabled seed germination at high temperatures. This effect was accompanied by a decrease in miR156 and an increase in miR172 levels.

The small RNAs miR159 (targeting transcripts of the myeloblastosis family genes MYB33, MYB65, MYB101) and miR160 (targeting transcripts of the gene ARF10) also impact on seed germination (Table 2). Changes in the levels of these miRNAs or in the sensitivity of the target transcripts alter the response of germinating seed to suppression of ABA biosynthesis [198,203,204]. Five further miRNAs (ath-miR8176, ath-miR851-5p, ath-miR861-3p, ath-miR158a-5p, and ath-miR779.2) showed the highest expression level during germination. Among these RNAs, miR851 might target the pentatricopeptide repeat (PPR) gene family, which is also expressionally upregulated during germination [165]. As some predicted targets of miR858a (MYB13, MYB65, and MYB93) are known as the regulators of germination, this RNA might also be involved in germination [165]. 

**Table 2:** The list of miRNAs involved in regulation of seed development and germination.

miRNAs	Target Gene(s)	Trait Affected	Species	References
miR160	*ARF10*	Germination, seedling growth	*A. thaliana*	[3]
miR858a	*MYB13, MYB65, MYB93*	Germination	*A. thaliana*	[4]
miR8176, miR851-5p, miR861-3p, miR158a-5p, miR779.2	Pentatricopeptide repeat (*PPR*) gene family	Germination	*A. thaliana*	[165]
miR156, miR172 (and high temperature)	*DOG1*	Dormancy, germination	*A. thaliana, L. sativa*	[202]
miR159	*MYB33, MYB65, MYB101*	Gibberellin, signaling pathway, germination	*A. thaliana*, *B. napus*	[1,2]
miR160, mi167	*ARF* *17, ARF8, ARF6, ARF10*	Auxin signaling pathway, germination	*A. thaliana*	[201]
7miR156	*BRI1*, *FUS3*, squamosa-promoter binding-like *(SPL)* gene family, etc.	Germination	*B. napus*	[201]
miR402	*DML3*	Germination	*A. thaliana*	[205]

To summarize, the epigenetic signals, such as the changes in DNA methylation, demethylation, histone post-translational modifications, and sRNA-related regulatory mechanisms, are the key modulators of seed development and the transition from seeds to seedlings. To date, the role of reversible DNA methylation and histone modifications accompanying seed germination is well-studied. However, specific miRNAs and their specific target genes are still mostly uncharacterized.

## 5. Conclusions

Due to complex temporal patterns of specific signals, deciphering the mechanisms behind the transition from seed to seedling represents a challenging task. Nevertheless, some exciting prospects for the future research in this area can be clearly seen. Thus, highly efficient comprehensive approaches to dissect these mechanisms at the epigenetic level will reveal gaps in our understanding of the transition from dormancy to germination. In this regard, the role of epigenetic modifications in the hormonal regulation of the transition from seed to seedling is of a particular interest. Obviously, detailed studies addressing the loss of desiccation tolerance during seed germination and aiming at identification of the involved genes, transcripts, proteins, and metabolites by means of comprehensive post-genomic techniques are still to be accomplished. Dynamics of chromatin, i.e., the transitions between its active and repressed states, are also poorly characterized in the context of seed germination, and the underlying molecular mechanisms remain mostly unknown. Physiological diversity of the seed to seedling transition is another issue to be addressed in the nearest future. Indeed, the mechanisms of seed dormancy and germination are mostly characterized for Arabidopsis as a model plant, whereas the crop plants remain to a high extent insufficiently addressed in this context. Thus, a comprehensive comparison of the mechanisms underlying the transition from dormancy to germination and from seed to seedlings in different species is strongly mandatory. The state-of-the-art methods of epigenomics research, such as bisulfite sequencing and 5-methylcytosine sequencing, might help to gain a deeper insight into the role of epigenetic variability in the formation of crop plant phenotype [206]. On the whole, the methods of genomics and post-genomic research provide a versatile instrument to probe the regulatory mechanisms behind the traits, promising in crop improvement programs. Finally, locus-specific modification of DNA methylation patterns by epigenome-editing tools might facilitate molecular breeding (epibreeding) of valuable crop plants.
